# Professional development for Indonesian elementary school teachers: Increased competency and sustainable teacher development programs

**DOI:** 10.12688/f1000research.156946.2

**Published:** 2025-04-28

**Authors:** Rasmitadila, Reza Rachmadtullah, Teguh Prasetyo, Megan Asri Humaira, Diah Andika Sari, Achmad Samsudin, Muhammad Nurtanto, Fitriyani, Rohimi ZamZam

**Affiliations:** 1 Department of Elementary School Teacher Education, Universitas Djuanda, Bogor, West Java, Indonesia; 2 Department of Elementary School Teacher Education, Universitas PGRI Adi Buana Surabaya, Surabaya, East Java, Indonesia; 3 Department of Elementary School Teacher Education, Universitas Djuanda, Bogor, West Java, Indonesia; 4 Department Early Childhood Teacher Education, Universitas Muhammadiyah Jakarta, Jakarta, Indonesia; 5 Program of Physics Education, Universitas Pendidikan Indonesia, Jawat Barat, Indonesia; 6 Department of Mechanical Engineering, Politeknik Negeri Jakarta, Jakarta, Indonesia; 7 Department of Elementary School Teacher Education, STKIP Kusuma Negara Jakarta, Jakarta, Indonesia; 8 English Department, Universitas Negeri Medan, Medan, North Sumatra, Indonesia

**Keywords:** Professional development, elementary school, professional teacher

## Abstract

**Background:**

Various problems, such as a shortage of teachers, especially in certain outlying and underdeveloped provinces, unequal distribution of teachers, low teacher competency, and mismatch between educational qualifications and scientific fields, as well as the demands of increasingly dynamic developments in science and technology, resulting in the need for professional development for elementary school teachers to improve teacher professionalism in Indonesia. These background as a basis for the government to create teacher professional education program for elementary school teachers continuously. It is hoped that it will be able to answer the educational problems facing the Indonesian nation. This research explores the opinions of elementary school teachers who have participated in teacher professional development activities through the in-service teacher professional education program.

**Methods:**

We collected data through a written survey of 24 elementary school teachers, as well as article documents relevant to the research topic. Data were analyzed using thematic analysis.

**Result:**

The research produced two main themes: increasing teacher competencies and sustainable teacher development programs. Increasing teacher competency related to social competence, pedagogical competence and professional competence. The sustainable teacher development program is related to the guaranteeing the instructional quality of post-program and multi-sectoral collaboration

**Conclusion:**

Developing teacher professionalism through teacher professional education program in elementary schools in Indonesia positively impacts teachers’ becoming professional teachers. Teacher professional education program is an indication of improving teacher professionalism, especially in developing teaching skills, improving the quality of instruction, teacher motivation and individual development in dealing with various problems in instruction in the classroom, especially in elementary schools, so that the instructional objectives that have been set can be achieved. The professionalism of elementary school teachers will improve if there are sustainable programs such as mentoring programs from the government, universities and parties that focus on improving the quality of education in Indonesia

## Introduction

Professional teachers in elementary schools are a significant need today to realize better quality education worldwide. Various elementary school teacher professional development (TPD) programs have been carried out in many countries to increase teacher competency in academic and non-academic fields, significantly improving student learning outcomes ( Gümüş and Bellibaş 2023; Avalos 2011). TPD is a continuous process of learning, reflection, and action to improve teacher knowledge and skills, resulting in better teaching practices and positively impacting student learning ( Luft and Hewson 2014; Singh, Rind, and Sabur 2021; Gafurov, Valeeva, and Kalimullin 2019). Several examples of TPD programs in elementary school which can be carried out through formal or non-formal means such as workshops, courses, seminars, degree programs, school observations, individual or collaborative research, peer-to-peer discussions or reading literature such as journal articles or theses are activities that teachers can choose and participate ( OECD 2009). Through holistic and sustainable professional development of teachers throughout their careers, teachers are expected to develop individual, social, pedagogical and professional skills to provide meaningful learning to students up to the highest level of education, including teachers in elementary schools.

The professional development of elementary school teachers will directly impact students who face major learning challenges to achieve learning achievement. Teachers must be able to renew and hone their abilities to increase student success, so there is a need for effective TPD programs. The most effective form of TPD in elementary schools is proven by teaching coaching and collaboration between teachers who are adapted to developing the teacher’s personality in a special program ( Porcell 2020; Botha and Herselman 2018; Lim, Juliana, and Liang 2020). TPD is an essential strategy in supporting the skills students need to prepare for further education in the 21st century ( Twining et al. 2013; Martinez 2022). In the future, students must be able to master more challenging content, problem-solving, communication, and collaboration so that teachers must be able to answer these challenges with more sophisticated and practical learning. Collaboration between colleagues, the exchange of knowledge, as well as complementary competencies motivating teachers to develop continuously will be a challenge in solving learning problems in the classroom, so more professional elementary school teachers are needed ( Van der Heijden et al. 2015; Bürgener and Barth 2018). The target for increasing teacher professionalism is, of course, the same as conditions in Indonesia.

The Indonesian government issued Law Number 14 of 2005 ( Legislation 2005) concerning teachers and lecturers, and Article 1 Paragraph 1 states that teachers are professional educators with the main task of educating, teaching, guiding, directing, training, assessing, and evaluating students in early childhood education through formal education, primary education, and secondary education. Based on this law, currently in its implementation, a program was held to increase teacher professionalism, namely the teacher professional education program (TPEP). This program was created not only as a legal regulation but also because of various problems related to the professionalism of elementary school teachers and the quality of education in Indonesia. Various problems, such as a shortage of teachers, especially in certain outlying and underdeveloped provinces, unequal distribution of teachers, low teacher competency, and mismatch between educational qualifications and scientific fields, as well as the demands of increasingly dynamic developments in science and technology, resulting in the need for professional development for elementary school teachers. For this reason, the Indonesian government, through the Ministry of Education, Culture, Research and Technology, organizes TPEP for elementary schools in collaboration with higher education, which has study programs for TPEP, which are divided into two types, namely in-service and pre-service programs ( Ministry of Education and Culture 2020). This program aims to ensure that elementary school teachers and prospective teachers carry out their professional duties responsibly and firmly, with discipline and mastery of the material, teach well, design learning, implement and evaluate learning, and develop themselves continuously.

This research explores elementary school teachers’ perspectives on teacher professional development through teacher professional education programs in Indonesia.

### Teacher professional development in various countries

TPD at the elementary school level refers to teacher development throughout their formal career by increasing knowledge, skills and competencies to create effective learning in the classroom. This professional development is the best practice in learning, such as handling students with various characteristics, involving parents and the community, including understanding legislation regarding the roles and responsibilities of teachers, and increasing teacher professionalism in the latest learning practices, including utilization of technology in learning. Through the development of teacher professionalism, it is hoped that there will be changes in the quality of learning, by: 1) changes in learning for the better, by implementing learning strategies effectively and efficiently; 2) support from all school members so that teachers continue to develop teacher potential, interests and talents so that they can achieve all school goals in the future; 3) teachers can develop themselves by understanding the characteristics of students from all cultural, economic and social backgrounds so that learning becomes better and of better quality; 4) teachers are given the opportunity to develop by applying an ever-evolving mindset, being able to face changes in classroom situations, so that they can train themselves in decision making and problem solving; 5) give opportunities for teachers to collaborate with other people, colleagues in developing critical ideas in solving problems in learning so that teachers have power creativity and innovation which continues to increase ( Tam 2015).

The TPD program has significantly provided benefits for improving the quality of learning through improving learning content to improve teachers’ teaching abilities in the classroom ( Gore and Rosser 2022; Richter et al. 2021). In addition, TPD has succeeded in developing a new framework for teachers by emphasizing collaborative learning and in-depth reflection on continuous learning that is real to class needs so that it can improve teaching practices for teachers in the classroom ( Sims et al. 2025). These changes and developments have an impact on student learning outcomes. For the TPD program to be successful and have a positive impact on teachers and students, training materials need to be emphasized on learning practices that are by class needs so that there needs to be innovation in learning that is responsive to the learning context and student needs, including adjusting learning with the use of learning technology that supports successful learning in the classroom ( Coppe et al. 2024; Ventista and Brown 2023).

Several TPD programs in elementary schools in several countries have resulted in several views. In China, curriculum reform places TPD as an integral part of teacher practice as formulated in policy and its implementation. TPD programs in elementary schools in China are directed at 21st-century skills with a focus on increasing competence through inquiry-based learning ( Kayange and Msiska 2016), one of which is research-oriented ( Wang 2018). The Chinese government has issued various policies related to elementary school teacher professional programs, including collaboration between schools and universities, lesson planning collaboration, sharing about teaching practices, exchange programs, coaching colleagues, and research activities such as action research and reflection. Even though this program has received criticism from various parties, such as the focus on assessment and student learning outcomes, the TPD program in China is by 21st-century learning conditions because it prioritizes inquiry-based, critical, self-motivated learning and collaboration between school teachers. This condition is oriented towards the teacher’s work environment and development technology ( Guo and Yong 2013).

Meanwhile, the experience of TPD programs in the Philippines states that there is a need for professional elementary school teacher programs because teachers are seen as implementers of the curriculum, and the lack of teacher training has resulted in the development of leadership in schools ( Alegado 2018). Another problem is the occurrence of emotional and work stress due to their work. This has implications on the spectrum of personal and professional development ( Bongo and Casta 2017), so there is a need for TPD. Through TPD, teachers in the Philippines can develop TPD activities, whether carried out by schools or other parties, which can be done by attending seminars and continuing education at a higher level to develop their personal and professional competencies. Teachers want to gain new knowledge and skills according to student development through seminar activities or training on topics with relevant issues such as pedagogy, classroom management, technology, and assessment. In Malaysia, to improve the quality of education and human resources, TPD was started in 1995 by a committee specifically in the Ministry of Education. A few recommendations include encouraging teachers to take courses, continue their education, and study abroad to gain knowledge and education, in addition to pre-service training and in-service training for primary and secondary school teachers ( Kabilan and Veratharaju 2013).

The European Commission’s policy emphasizes that TPD, including in elementary schools, consists of a 3-phase model: initial teacher education, induction (for new teachers, 3-5 years after graduation), and in-service teacher education to support teachers’ long-term career development. With this policy, European member states agree to continuous teacher education to ensure that initial teacher education, early career support, and further professional development are organized, regular, and highly quality (Commission of the European Communities 2007). Specifically in Finland, TPD programs are based on goal-oriented teacher leadership, school development, and quality teaching through collaboration and colleague interaction. TPD can be done through training and research. For example, in-service training in Finland is based on training time and short courses offered to teachers in a holistic and integrated manner so that teachers can design school-based projects and their development related to school development. Successful professional development of teachers in Finland also exists in collaboration with parents as external parties who provide full support, especially at the beginning of their career as teachers ( Niemi 2015).

Meanwhile, in the United States, most states require professional development and continuing education. Locally, schools provide opportunities for teachers to continue their education through professional development activities hosted by the school district ( Wei, Darling-Hammond, and Adamson 2010). However, no national policy determines the content and methods of professional development programs. Development program activities can be carried out using short-term workshops, training, and master teacher programs that provide special training and financial incentives to participating teachers.

### Teacher professional development in Indonesia

Elementary school teachers are educational personnel who will provide students with basic skills that will continue at the next level of education. For this reason, students’ best achievements will be significantly influenced by the professionalism of elementary school teachers in implementing all the skills they have. The Indonesian government pays special attention to the development of teacher professionalism, including elementary school teachers. This development commitment was issued by Law number 14 of 2005 in Article 1 Paragraph 1 concerning teachers and lecturers, which states:

“Teachers are professional educators with the main task of educating, teaching, guiding, directing, training, assessing and evaluating students in early childhood education through formal education, basic education and secondary education.”

This law explains that the duties of teachers, including elementary school teachers, are very complex and focused on improving the quality of education in Indonesia. However, until now, the quality of education in Indonesia still needs to improve, and some of the problems are due to the low professionalism of teachers. This low level of professionalism is demonstrated, among other things, by the low level of teacher competence, such as pedagogical competence ( World Bank 2015), even though the government has implemented various policies and increased educational investment to improve education quality. These results continue to be the basis for the government to create professional development programs for elementary school teachers continuously. The TPD program at elementary school began in 1980 with a teacher work consolidation program in the form of in-service training, which aimed to train teachers to practice student-centred instructional methods. In 1993, the Indonesian government created a similar program for elementary school teachers through teacher working groups. With the Teacher and Lecturer Law in 2005, the government developed a teacher certification program with a teacher professionalism program in teacher education and professional training. Some of these programs still need to improve classroom learning quality ( Revina et al. 2020).

The government of Indonesia, through the Ministry of Education, Culture, Research and Technology, continues to develop professional teachers in the form of TPEP for elementary school teachers aimed at being designed systematically and by applying quality principles from the selection, learning, and assessment processes to competency tests, the aim is to develop future teachers who are professional and able to produce outstanding graduates. These graduates should be competitive, have strong character, and possess a love for their country. Additionally, the hope is that they will be able to address the educational challenges that the Indonesian nation is facing. The TPEP program is held after an undergraduate or applied undergraduate program, which demands different competency standards for graduates from an applied bachelor’s or bachelor’s program. TPEP graduate competency standards, which include attitudes, knowledge, and skills, are stated in learning outcomes. The TPEP is divided into two types, namely pre-service and in-service. Pre-service is an educational initiative held after completing an undergraduate or applied undergraduate program, open to Bachelor graduates from various backgrounds, both educational and non-educational, who are interested in becoming teachers ( Ministry of Education and Culture 2020).

This program aims to obtain a special educator certificate in early childhood, primary, and secondary education. The benefits of pre-service are: 1) obtain an educator certificate; 2) improve pedagogical, social, professional and personality competencies to start a career as a professional teacher; 3) obtain recognition as a professional teacher. Meanwhile, in-service programs target elementary teachers currently active in educational institutions’ teaching profession. This program’s main focus is to improve teachers’ qualifications and abilities, aiming to become professional and superior educators in quality. Through this program, teachers can apply innovative teaching methods, encourage creativity, and improve student learning outcomes in elementary schools. This program also aims to increase teacher professionalism and provide a better understanding of the duties and responsibilities of a teacher.

In this research, we specifically explain in-service TPEP for elementary school teachers who have various materials and competencies related to the field of education, including pedagogy, educational psychology, educational management, and teacher professionalism. TPEP activities in this position are carried out over almost two months and take the form of 5 learning activities: 1) deepening the material in the field of expertise and pedagogy that will be taught; 2) development of learning tools; 3) practical field experience. The total credits taken in this program are 12 credits. In deepening the material, teachers will learn online for 5 credits. Learning activities aim to help teachers strengthen their understanding of professionalism, pedagogy, and subject matter by utilizing information and communication technology in online and independent learning through learning management systems (LMS) and other sources ( Ministry of Education and Culture 2020).

To ensure teachers’ professionalism, it’s crucial for them to have an in-depth understanding of learning theories, educational psychology, sociocultural influences, social construction, and diversity. Expanding pedagogical expertise involves the careful planning, execution, and assessment of educational activities. The development of learning tools encompasses a 3-credit program aimed at assisting elementary school educators in creating effective learning tools through workshops that leverage their deep professional knowledge, pedagogical skills, and relevant areas of study. This includes curriculum analysis, designing educational activities, and crafting learning assessments using the Technological Pedagogical and Content Knowledge (TPACK) approach within the framework of Industry 4.0. The objective is to design educational activities and assessments that integrate critical thinking, creative thinking, reflective thinking, and decision-making skills through inquiry-based learning activities. Field experience practice activities with a credit load of 4 facilitate teachers to develop professional skills as teachers whose main task is to educate, teach, guide, direct, train, assess and evaluate students which is carried out as teaching practice.

This activity also facilitates teachers in carrying out non-teaching tasks in class and school administration, developing co-curricular and extra-curricular activities, and participating in other school activities. After implementing the three main activities, teachers will carry out performance exams and knowledge exams to measure and determine teacher performance achievements in planning, implementing and evaluating learning, as well as measuring and determining the level of teacher mastery of the field of study and pedagogy and deserving of educator certification.

## Methods

To explain the teacher’s professional development in elementary schools, we carried out a case study approach to allow researchers to obtain and examine data in a context or phenomenon in two ways. The first way was carried out using a written survey of 24 teachers who had implemented the in-service TPEP by the Ministry of Education, Culture, Research and Technology of the Republic of Indonesia in 2023. We also used documents from journals relevant to TPD, especially in Indonesia. Before participating in the study, participants were given complete information about the aims and procedures and provided written informed consent.

### Context and participants

This research uses a purposive sampling strategy. The research location consists of 24 elementary schools in 3 provinces in Indonesia, namely Jakarta, West Java and East Java. The selection of elementary schools was based on the criteria of public and private schools with teachers who had completed a TPEP for almost two months. These schools are also for teachers who participate in the TPEP, where we are as tutors (TPEP’s educators). Meanwhile, teacher selection is based on the following criteria: 1) teachers with at least two years of teaching experience; 2) teachers who have participated in the in-service TPEP for almost two months. The research participants comprised 24 people (21 women and three men). They have teaching experience ranging from 2-30 years in elementary schools. They all had formal degrees (bachelor’s) before entering teaching careers. These teachers have been included in the criteria to see the impact of the TPEP that they have implemented, and are related to their teaching experience over a sufficient period of time, so that they can be used as participants in the research.

### Data collection and analysis

Data was collected in 2 ways; the first was using a survey with the help of Google Forms, which contained open questions consisting of 5 questions related to the implementation of the TPEP. Data collection was carried out over five days by providing a Google Form link to 24 teachers, and we monitored all teachers to ensure that they had filled in the link. The second way is to collect article documents that are relevant and specific to the results of professional teacher education programs using Google Scholar in Indonesia. We found 8 articles from national journals accredited by the Ministry of Education, Culture, Research and Technology of the Republic of Indonesia (Sinta) and DOAJ, Copernicus, Google Scholar, Scopus and Web of Science. The topics we collected from journal articles are related to TPD through TPEP, including the program’s impact, influence and effects. After data was collected from both data types, we began processing the data for data analysis. Data were analyzed using thematic analysis. We identify, evaluate and make the main themes revealed by researchers. In this analysis, we used an inductive method, coding both types of data, and to make it easier to create categorization and themes

### Ethical consideration

The research and community service department of Universitas Djuanda has approved this research with the certificate number: 341/LPPM/K-X/X/2024 on February 8 ^th^ 2024. The researcher gave a letter of approval has also been given by the researcher to all respondents. Written consent to participate from the respondent was obtained in accordance with document 01/K-X/IX/2024 on January 24 ^th^ 2024. Respondents gave their consent without force from anyone. Subsequently, in order to protect the rights and privacy of the respondents, all forms of data acquired will remain confidential.

## Results

**Figure 1.**
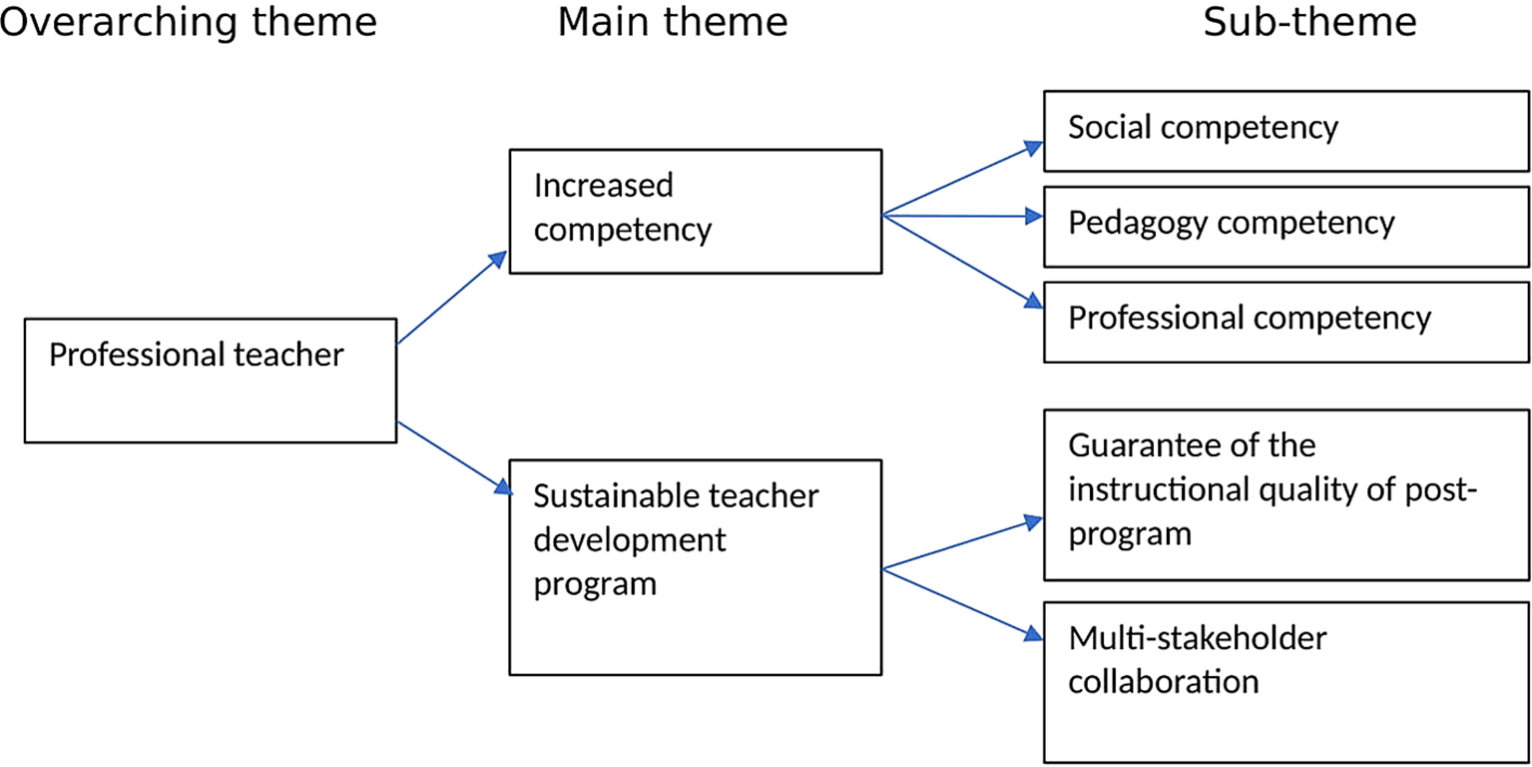
Theme of data analysis.

This study seeks to explore TPD through TPEP by examining elementary school teachers’ perspectives. For this purpose, teachers’ experiences related to professional development practices in the elementary school environment are explored. The themes resulting from data analysis consist of 2 main themes, namely increased competency and sustainable teacher development programs by Figure 1:

### Increased competency

Increasing competency is related to increasing teacher ability and competency standards by objectives, job demands, and science and technology. The results of data analysis using interviews and journal articles on the theme of increasing competence consist of the sub-themes of social competence, pedagogical competence and professional competence. According to the findings regarding social competence, the teacher’s perspective consists of communication and adding colleagues. Communication between teachers and program participants increases motivation to carry out the program more enthusiastically at each stage, such as getting input using learning models, media, and assessments. With a grouping system consisting of 10-12 teachers from different schools, the teachers provide input and opinions to improve teacher competence during the program. The pattern of the program implemented by teachers increases bounding and cooperation so that they have friends who generally have the same problems, indicating that this program adds colleagues who support each other in improving competence. Several teachers explain this opinion:

“For me, this program adds friends who can solve problems in class which are generally the same as how to choose a instructional model that suits our class.”

“I practice my communication with my group friends, so that I gain experience in providing services to my students in class.”

For the sub-theme of pedagogical competence, the teachers’ opinions stated that with this program, they gained an understanding of meaningful learning, the use of learning tools, and the selection of learning strategies. According to teachers, understanding meaningful learning is achieved by mapping problems in the classroom. They can design learning according to student’s needs and choose methods and learning media that suit the characteristics of their students so that students get meaningful learning. One teacher explained this:

“With this program, I am better prepared to prepare classes according to the characteristics of my students, so that learning is more meaningful.”

The use of learning tools is also one of the findings that teachers benefit. Teachers think that during the training program, making learning tools such as lesson plans that are appropriate to learning outcomes, learning reflections and examples of good practices that can be implemented in the classroom has a positive impact on teachers in improving learning designs in their classes, as well as understanding shortcomings when teaching. This opinion was explained by one of the teachers:

“I came to understand how to create learning tools such as lesson plans and reflections so that I understand my shortcomings in teaching.”

The following finding regarding the selection of learning strategies according to teachers is how they determine learning strategies, such as using learning methods, using learning media that they can make manually or using technology. These learning steps are appropriate to their learning methods, to assessments that do not only use cognitive aspects but can assess all aspects (cognitive, affective, psychomotor). Choosing a learning strategy, according to the teacher, provides an opportunity for teachers to explore several varied learning methods that they have not only used so far, but by looking at their other colleagues, it becomes input for them in choosing an effective learning method for their class. This opinion was expressed by one of the teachers:

“In this program, I was taught about choosing effective and best instructional methods according to the conditions of my class. I came to understand that there are many instructional methods and instructional media that I can use in my class.”

In the sub-theme of professional competence, findings consist of professional teachers, knowledge, understanding of technology, experience, and problem-solving solutions. Findings about professional teachers: Teachers hope they become more professional by better understanding education’s meaning through improving learning after the program. Meanwhile, science is related to additional new knowledge and broader thinking. Teachers get many benefits, especially expanding knowledge, such as the latest instructional methods from tutors and problem-solving methods that are always connected to effective thinking skills so that teachers gain broader insight into learning. One teacher stated:

“Through this program, I have gained many benefits, especially increasing my knowledge, my insight has increased, especially how to solve the problems that I have been facing.”

Findings regarding understanding technology are related to the use of technology, such as online meeting applications and operating applications for instruction, which aim to help teachers carry out tasks given by tutors and academic and non-academic tasks, such as online meeting applications. Because the teachers in this program are teachers in the senior category (mostly over 50 years old), this is a good achievement, especially in operating the internet and computer programs, which can support the smooth running of the program, as well as solving learning problems by using technology-based instructional media. The results of the article used in this research also showed that using technology in learning is one of the benefits of this professional teacher program. Meanwhile, the opinion of one teacher:

“I was able to create my assignments using existing technology (Canva, AI, Infocus), which I rarely used in learning previously.”

From the experience findings, the teacher believes that through professional development, teachers have added significant experiences for teachers. For example, teachers gain experience in designing instruction according to the characteristics of their students, which they have yet to do using initial stages such as mapping learning styles, class problems, especially subjects faced by students, and the real needs of students by predetermined learning outcomes. So far, they have always used instructional methods according to the teacher’s wishes, not the student’s (teacher-centred). Meanwhile, the findings regarding problem-solving in this subtheme are the methods teachers use related to learning difficulties, not achieving learning goals, and handling students with many characteristics. Through this program, teachers get many ways and choices in solving learning problems in the classroom, especially students’ learning difficulties. Teachers are starting to be able to design and choose instructional methods and media in the form of lesson plans for a limited duration or learning time, but with continuous practice, getting feedback from tutors, and revising them again so that they get maximum lesson plans have provided an in-depth experience that teachers can use when teaching in their classes. One teacher’s opinion:

“In this program, I get attention and service from tutors in designing lessons, for example. We will design a lesson plan, revise it and get the best feedback that we can practice in our class according to the characteristics of the students.”

### Sustainable teacher development program

The theme of the sustainable teacher development program is related to the two sub-themes of guaranteeing the instructional quality of post-program and multi-sectoral collaboration. The sub-theme guarantee of the instructional quality of post-program is related to monitoring the quality of learning and special teacher programs. Findings regarding monitoring the quality of learning are after the TPEP. In several articles analyzed, monitoring the quality of instruction after the program is necessary because it affects the overall quality of education. The teacher’s opinion states that there must be a special program after the program so that the teacher’s enthusiasm and motivation to improve the quality of instruction is higher. Teachers also think that the government should be able to provide scheduled special programs, for example, teacher meetings in certain periods on certain topics that can maintain the quality of teacher learning, information on instructional methods, and the latest learning applications. One teacher stated:

“I hope that there will be a scheduled follow-up program that can be facilitated by the education department, for example, which can give teachers the opportunity to update their knowledge regularly, so that the quality of instruction can continue to improve.”

Meanwhile, the findings of the multi-sectoral collaboration subtheme relate to the need for collaboration between the government, educational institutions (universities), schools, and the community in supporting professional teacher education programs, especially post-program. The government must be able to embrace all parties to be involved in improving the quality of education. Support from all sectors can improve educational services because the quality of instruction will improve due to the implementation of professional teacher programs in elementary schools. With the involvement of all sectors and all parties, for example, parents can evaluate the quality of instruction through the school committee regarding the learning outcomes students achieve each semester.

## Discussion

In this research, we conducted a written survey of teachers who had implemented TPD through TPEP at elementary schools, and we used article documents relevant to the research topic. We analyzed both types of data based on theoretical background and research findings in teacher professional development. The development of teacher professionalism at the elementary school level, organized by the Ministry of Education, Culture, Research and Technology of the Republic of Indonesia, is a mandatory program for teachers through the TPEP. This research shows that it is essential to develop teacher professionalism in scheduled programs included in the national and other informal programs. In particular, TPD through TPEP can improve teacher competency, which is expected to be implemented sustainably in other formal and informal programs. Overall, we succeeded in finding a theme for a professional teacher with two main themes, which emphasized competency and sustainability programs for teachers so that the quality of instruction is maintained well after the professional teacher program. We identified two the main themes that are very important for achieving teacher professionalism, especially in elementary schools in Indonesia.

*First*, increasing teacher competency is the main requirement for teachers in solving instructional problems in elementary school classes. In the TPEP, there are four standard competencies that teachers must have, namely personal, professional, pedagogical and social competencies. We found only the three most dominant competencies in this research, namely social, pedagogical and professional competencies. Social competence refers to the teacher’s ability to communicate and get along with colleagues, education staff, students, parents, and the community. In this program, all activities are done in one group with the same goal. Therefore, communication between teachers and tutors, a sense of having the same goals, and helping each other academically and non-academicly greatly impact teachers’ achievements ( Atawolo and Hartoyo 2023). The results of the instructional design that has gone through a process with colleagues and tutors in providing constructive input also make it easier for teachers to implement it with students in the classroom. Good communication with colleagues motivates teachers to communicate and interact well with students ( Robinson 2022; Iurea 2015).

Increasing pedagogical competence is a teacher’s ability to understand students, design and implement instruction, develop students, and evaluate student learning outcomes to actualize their potential ( Shernoff et al. 2017; Caena and Redecker 2019). In this TPEP, we see that the process in each stage carried out by teachers begins with mapping the instructional problems in the classroom for each teacher. Teachers must systematically understand the problems that have been obstacles to achieving learning goals with various student characteristics. The problems found are analyzed and included in the instructional planning, which will then be implemented in the class, and the tutor will evaluate their achievements. The steps in each stage of the program, such as deepening the material, developing learning tools, and field practice (classroom practice), are the latest knowledge and insights obtained by teachers. With this experience, teachers gain ways to design effective instruction, understand students’ potential and understand the strengths and weaknesses of teachers they need to learn. Teachers can develop the latest instructional methods according to class characteristics, using instructional media and technology to make instruction more interesting and enjoyable for students ( Tang and Austin 2009; Haleem et al. 2022). In contrast, all students can achieve learning achievements. For example, teachers in this program use problem-based learning (PBL) methods to solve learning problems in the classroom. Teachers learn about the benefits and objectives of PBL so that learning outcomes exceed the expectations that teachers have previously lacked.

Meanwhile, increasing professional competence is a teacher’s mastery of learning material broadly and deeply, both curriculum material, subjects and the substance of knowledge that covers the instructional material ( Kartini et al. 2020; Davis, Janssen, and Van Driel 2016). With an academic background - not from an elementary school teacher program - the TPEP provides new knowledge for teachers, as provided by university tutors with expertise and research results relevant to teacher professional development. Tutors and teachers discuss competency standards that students must achieve comprehensively from cognitive, affective and psychomotor aspects, which teachers and colleagues rarely do. This becomes input for teachers in developing lesson materials to be more systematic, understanding the relationships between materials, sorting materials from simple to complex, and even creating additional teaching materials if students need special time to understand certain materials.

*Second*, the sustainable teacher development program is the hope of teachers that the elementary school TPEP is only one way for the government to increase teacher professionalism in Indonesia. We see the need for a similar program that is sustainable and scheduled to renew the competence of teachers who have taken part in this teacher professional development. Professional development of elementary school teachers is an activity that increases teacher motivation in teaching and enthusiasm for improving the quality of education, especially in Indonesia ( Sumantri and Whardani 2017; Bernat 2010). Routine activities initiated by the education department, school community, or teacher community with formal and informal beneficial activities, such as discussing literature or discussing the latest instructional methods with colleagues, can maintain teacher motivation so that they remain enthusiastic about improving their personal skills. Apart from that, attending seminars and educational workshops organized by the government or other parties can also maintain the positive impact that teachers have received after the TPEP. For this reason, it is necessary to involve all parties in developing the professionalism of elementary school teachers ( Fuadi, Nasution, and Wijaya 2023; Agustina, Suriansyah, and Asniwati 2021). The government can collaborate with other parties, such as the private sector or other bodies, to implement advanced programs that motivate teachers to become more professional.

Based on our research findings, we explain that TPEP is one way to improve teacher competence through a continuous teacher professional development program. This program is relevant to learning practices for teachers as long-life learning must be in accordance with experience and needs and based on solving real problems in the classroom. Several countries that have implemented programs similar to TPEP, such as Finland, implement structured and continuous teacher professional development programs based on research results and conduct teacher training throughout their careers so that teacher competence becomes the primary focus in learning, makes Finland one of the countries with the best education systems in the world ( Ostinelli and Crescentini 2024; Niemi and Lavonen 2020). In addition, Canada also carries out a program similar to TPEP by building a collaborative culture and focusing on reflective activities and teamwork ( Jäppinen, Leclerc, and Tubin 2016). Both examples of the implementation of the TPEP program and its success, in contrast, of course, some weaknesses or obstacles must be considered by each country so that TPEP becomes one of the paths that can be used to improve teacher professional development programs. Some obstacles or weaknesses of TPEP practices, for example, in Indonesia, are limited resources, both budget, and infrastructure such as facilities, supporting technology with a large number of teachers and low teacher competence requiring a lot of funding sources, so there are still limitations in implementing TPEP evenly in all provinces ( Permatasari 2024; Rahmat and Gunawan 2022). This is still a significant obstacle in implementing the TPEP program in many developing countries. Another problem is the government’s commitment to the TPEP program because changing priorities in a country cause the TPEP program to no longer be a priority, so increasing teacher competence is slower ( Ainscow et al. 2025; Fatimah, John, and Hasibuan 2025). This condition impacts changes in commitment, motivation, and uneven quality of teacher training. If the TPEP program is implemented with proper planning based on long-term targets and national standards that are oriented towards improving teacher competence, it can become a mainstay program for a country, including Indonesia, because, in theory, it has proven to be relevant and is practiced by many countries.

Specifically, the TPEP program is a very real practice of implementing relevant and contextual learning theories that use training materials that are appropriate to teacher needs ( Uribe-Zarain, Hellman, and Bell 2025), based on experience and in-depth self-reflection of teachers so that they can improve teacher competence ( Hindaryatiningsih et al. 2025). Through TPEP, teachers are trained to design learning according to the needs and problems in the classroom so that the teacher successfully achieves the learning objectives set by the teacher and impacts student learning achievement ( Albay and Eisma 2025). The TPEP program aligns with the Competency-Based Professional Development Theory, which is based on increasing experience and developing more specific competencies through collaborative learning, teamwork, mentoring, in-depth reflection, and peer observation ( Sun 2025; Zulfizar 2025). Through best practices carried out by teachers, they understand the shortcomings and advantages of the teaching they have done so far so that these results can be a basis for teachers to improve the quality of learning to be better.

## Conclusion

Developing teacher professionalism through TPEP in elementary schools in Indonesia positively impacts teachers’ becoming professional teachers. Increasing teacher competence is an indication of improving teacher professionalism, especially in developing teaching skills, improving the quality of instruction, teacher motivation and individual development in dealing with various problems in instruction in the classroom, especially in elementary schools, so that the instructional objectives that have been set can be achieved. The professionalism of elementary school teachers will improve if there are sustainable programs such as mentoring programs from the government, universities and parties that focus on improving the quality of education in Indonesia.

## Ethics and consent

The research and community service department of Universitas Djuanda has approved this research with the certificate number: 341/LPPM/K-X/X/2024 on February 8 ^th^ 2024. The researcher gave a letter of approval has also been given by the researcher to all respondents. Written consent to participate from the respondent was obtained in accordance with document 01/K-X/IX/2024 on January 24 ^th^ 2024. Respondents gave their consent without force from anyone. Subsequently, in order to protect the rights and privacy of the respondents, all forms of data acquired will remain confidential.

## Data Availability

Figshare: ‘Professional development for Indonesian elementary school teachers: Increased competency and sustainable teacher development programs’ Doi: https://doi.org/10.6084/m9.figshare.27141483.v3 ( Rasmitadila et al., 2024a) This project contains the following underlying data:
• Data Sheet-TPEP (2).numbers Data are available under the terms of the Creative Commons Attribution 4.0 International license (CC-BY 4.0). Figshare: Journal contribution. Doi: https://doi.org/10.6084/m9.figshare.27225480.v1 ( Rasmitadila et.al., 2024b) This project contains the following extended data:
• Journal Contribution.docx Data are available under the terms of the Creative Commons Attribution 4.0 International license (CC-BY 4.0).
