# Supplementary material for: Professional development for Indonesian elementary school teachers: Increased competency and sustainable teacher development programs
Source: F1000Res. 2025 Aug 6;13:1375. Originally published 2024 Nov 18. [Version 3] doi: 10.12688/f1000research.156946.3 (PMC12419144; doi:10.12688/f1000research.156946.3)
Supplement: Peer review history

# Reviewer report: Reviewer response for version 3

**DOI:** 10.5256/f1000research.186045.r406259

**Published:** 2025-09-03

**Authors:** Nurul Hidayati Rofiah

**Affiliations:** 1 Universitas Ahmad Dahlan, Yogyakarta, Special Region of Yogyakarta, Indonesia

This article makes a valuable contribution by examining Indonesian elementary school teachers’ experiences with the Teacher Professional Education Program (TPEP) and highlighting two central themes: improving teacher competencies and ensuring sustainable teacher development. The study is timely and relevant, given the urgent need to enhance teacher professionalism to address challenges in education quality across Indonesia.

 While the discussion identifies important implications, it could be strengthened by explicitly addressing the strengths and weaknesses of the study, and by offering clearer directions for future research and policy implementation.

Is the work clearly and accurately presented and does it cite the current literature?

Yes

If applicable, is the statistical analysis and its interpretation appropriate?

Not applicable

Are all the source data underlying the results available to ensure full reproducibility?

Partly

Is the study design appropriate and is the work technically sound?

Yes

Are the conclusions drawn adequately supported by the results?

Yes

Are sufficient details of methods and analysis provided to allow replication by others?

Yes

Reviewer Expertise:

Elementary teacher education, inclusive education, disability inclusive disaster risk reduction, religion

I confirm that I have read this submission and believe that I have an appropriate level of expertise to confirm that it is of an acceptable scientific standard.

# Reviewer report: Reviewer response for version 2

**DOI:** 10.5256/f1000research.180838.r398100

**Published:** 2025-07-29

**Authors:** Minsih Minsih

**Affiliations:** 1 Universitas Muhammadiyah Surakarta, Surakarta, Indonesia

This article has a novel value that has a significant impact on the teacher professional development (TPD) program in Indonesia. The research conducted illustrates the need for a sustainable program for teachers to have a global picture of the importance of teacher professional development everywhere. The main gaps or problems in Indonesia have been explained in detail and focus, so that the teacher professional education program (TPEP) is one program that should be implemented by the Indonesian government. The research method used a survey and analyzed using thematic analysis. In this data analysis process, it is advisable to explain the steps of the thematic analysis, and the researcher's involvement in data validation. The discussion should also connect each finding with theoretical frameworks or previous literature, for example, teacher competency development theory, the TPD model, or adult learning theory (andragogy). Furthermore, the importance of a sustainable program in the form of its concrete implementation is emphasized.

Is the work clearly and accurately presented and does it cite the current literature?

Yes

If applicable, is the statistical analysis and its interpretation appropriate?

Yes

Are all the source data underlying the results available to ensure full reproducibility?

Yes

Is the study design appropriate and is the work technically sound?

Yes

Are the conclusions drawn adequately supported by the results?

Yes

Are sufficient details of methods and analysis provided to allow replication by others?

Partly

Reviewer Expertise:

Elementary School, Teacher Training, and Inclusive Education

I confirm that I have read this submission and believe that I have an appropriate level of expertise to confirm that it is of an acceptable scientific standard.

# Response

**Published:** 2025-08-02

**Authors:** Rasmitadila Rasmitadila

**Affiliations:** Universitas Djuanda, Indonesia

Dear Dr. Minsih,

 Thank you for the valuable and expertise comments you provided for this articles.  We have added the statements based on you comments, such as:

 1. Steps of data analysis: We added the specific steps for the data analysis, as well as a validation step by each researcher (p. 9)

 2. Connect it to several theories: We added statements about the theory of andragogy, and TPD at the Discussion section (p. 15)

# Reviewer report: Reviewer response for version 1

**DOI:** 10.5256/f1000research.172335.r357153

**Published:** 2025-02-06

**Authors:** Yifan Ou

**Affiliations:** 1 University of Antwerp, Antwerp, Belgium

**Strengths**

**Rich and Relevant Research Background**:

 The article provides a detailed overview of the current state and significance of Teacher Professional Development (TPD) on a global scale, with a particular focus on Indonesia's Teacher Professional Education Program (TPEP). The background information is comprehensive, allowing readers to quickly understand the necessity and urgency of the research.

 It emphasizes the crucial role of primary school teacher professional development in improving student learning outcomes and addressing the challenges of 21st-century education, aligning with current hot topics in the field of education.

**Rigorous Research Methodology**:

 The study employs a case study approach, combining surveys and literature analysis, with diverse data sources that support the research conclusions from multiple perspectives.

 Thematic analysis is used to process the data, which is a scientific and rational method that helps extract valuable information from a large amount of data.

 The research clearly outlines ethical considerations, including obtaining approvals and informed consent from participants, in line with basic academic standards.

**Systematic and Comprehensive Research Content**:

 The article offers a detailed comparative analysis of the current state of primary school teacher professional development across different countries, providing an international perspective and highlighting the uniqueness and借鉴意义 of Indonesia's TPEP.

 It delves into the specific content, goals, implementation methods, and participants of TPEP, covering multiple aspects of teacher professional development.

**Inspiring Research Findings**:

 Two main themes are identified—enhancing teacher competence and sustainable teacher development programs—which offer valuable insights for primary school teacher professional development in other countries and regions.

 The rich and diverse feedback from teachers reflects the actual impact of TPEP on their professional growth, enhancing the credibility of the research.

 -------------------------

**Weaknesses**

**Limited Research Sample**:

 The study only includes 24 primary school teachers from three provinces in Indonesia. The small sample size and limited geographical scope may not fully represent the situation of primary school teachers nationwide, restricting the generalizability of the research findings.

**Methodological Limitations**:

 Although surveys and literature analysis are used, the surveys are conducted solely through Google Forms, which may limit the ability to gain in-depth insights into teachers' complex views and emotional experiences.

 There is a lack of direct observation or case analysis of classroom practices, making it difficult to comprehensively assess the direct impact of TPEP on teachers' actual teaching behaviors and student learning outcomes.

**Need for Deeper Analysis**:

 The discussion section provides a relatively brief analysis of the research findings, lacking in-depth theoretical exploration and comparative analysis with other studies. The reasons for the success or shortcomings of TPEP are not thoroughly examined.

 The specific implementation suggestions for sustainable teacher development programs are not detailed enough, lacking actionable plans and predictions of expected outcomes.

**Insufficient Timeliness and Comprehensiveness of Literature Review**:

 Some of the cited literature is outdated, which may fail to cover the latest research findings and trends.

 The analysis of primary school teacher professional development programs in other countries is not in-depth enough, lacking a summary and comparison of lessons learned from different countries. This makes it difficult to highlight the unique advantages and areas for improvement of Indonesia's TPEP.

 ---------------------------------

**Revision Suggestions**

**Deepen Theoretical Analysis**:

 Strengthen the theoretical interpretation of research findings in the discussion section, integrating relevant domestic and international theories and the latest research results to deeply analyze the successful experiences and shortcomings of TPEP.

**Improve Literature Review**:

 Update and supplement the latest literature, especially cutting-edge international research on primary school teacher professional development, to enhance the academic and forward-looking nature of the study.

**Overall**, the article has certain academic value and potential for getting approved for indexing, but there is room for improvement in terms of research depth, breadth, and methodology.

Is the work clearly and accurately presented and does it cite the current literature?

Partly

If applicable, is the statistical analysis and its interpretation appropriate?

Partly

Are all the source data underlying the results available to ensure full reproducibility?

Partly

Is the study design appropriate and is the work technically sound?

Partly

Are the conclusions drawn adequately supported by the results?

Yes

Are sufficient details of methods and analysis provided to allow replication by others?

Partly

Reviewer Expertise:

psychology, communication and mental health

I confirm that I have read this submission and believe that I have an appropriate level of expertise to confirm that it is of an acceptable scientific standard, however I have significant reservations, as outlined above.

# Response

**Published:** 2025-04-17

**Authors:** Rasmitadila Rasmitadila

**Affiliations:** Universitas Djuanda, Indonesia

Dear Yifan Ou,

 Thank you for allowing us the opportunity to submit a revised draft of the manuscript “ Professional development for Indonesian elementary school teachers: Increased competency and sustainable teacher development programs” for publication in the F1000Research.

 We appreciate the time and effort that you dedicated to providing feedback on our manuscript and are grateful for the insightful comments and valuable improvements to our paper. We have revised the article based on your suggestions. Please see below for a point-by-point response to the reviewers’ comments and concerns: 

 1. Deepen Theoretical Analysis: We added the theory about the TPEP that is relevant to our research findings in the discussion section (p.14)

 2. Improve Literature Review: We added the latest research about teacher professional development in the literature review section (p.5)
